# Investigations into Isoniazid Treated* Mycobacterium tuberculosis* by Electrospray Mass Spectrometry Reveals New Insights into Its Lipid Composition

**DOI:** 10.1155/2018/1454316

**Published:** 2018-06-19

**Authors:** Rahul Pal, Saif Hameed, Varatharajan Sabareesh, Parveen Kumar, Sarman Singh, Zeeshan Fatima

**Affiliations:** ^1^Amity Institute of Biotechnology, Amity University Haryana, Gurugram, Manesar 122413, India; ^2^Advanced Centre for Bio Separation Technology (CBST), Vellore Institute of Technology (VIT), Vellore, Tamil Nadu 632014, India; ^3^Division of Clinical Microbiology and Molecular Medicine, Department of Laboratory Medicine, All India Institute of Medical Sciences, New Delhi 110029, India

## Abstract

Many of the earlier studies involving the effect of isoniazid (INH) treatment have solely focused on the fatty acyl (FA) category of* Mycobacterium tuberculosis* (MTB) lipids. This motivated us with the major interest to examine the impact of INH on various other categories of MTB lipids. Towards this, we chose to interpret our mass spectral data (LC-ESI-MS) by a standalone software, MS-LAMP, in which “Mtb LipidDB” was integrated. Analysis by MS-LAMP revealed that INH treatment can alter the composition of “glycerolipids (GLs)” and “glycerophospholipids (GPLs)” categories of MTB lipids, in addition to the variations to FA category. Interpretation by “MycoMass” database yielded similar results as that of Mtb LipidDB, except that significant alterations to polyketides (PKs) category also were observed. Probing biosynthetic pathways of certain key lipids belonging to any of GLs, GPLs, and PKs categories can be attractive target(s) for drug discovery or can be useful to identify means to overcome drug resistance or to obtain insights into the causal factors of virulence. To the best of our knowledge, this is the first report hinting at the influence of INH on GLs, GPLs, and PKs of MTB.

## 1. Introduction

Tuberculosis (TB) still remains serious illness globally and the foremost common cause for death among patients with AIDS [1].* Mycobacterium tuberculosis* (MTB) the causal organism of TB is tough to control in the lieu of emergence of resistant strains inflicting multidrug resistance (MDR). MDR-TB attributes to isoniazid (INH) and rifampicin (RIF) resistance, while extensively drug-resistant tuberculosis (XDR-TB) involves extra resistance to fluoroquinolones along with one of the three injectable medications, kanamycin, amikacin, or capreomycin [2]. One of the strategies to combat the MDR phenomenon against MTB is to discover novel targets for currently used anti-TB drugs such as INH, RIF, and ethambutol (EMB).

Isonicotinic acid hydrazide or commonly known as isoniazid (INH) is one of the most significant anti-TB first-line drugs, which impede mycolic acid synthesis to kill actively growing intracellular and extracellular tubercle bacilli [3]. INH targets InhA (EC 1.3.1.9, enoyl-[acyl-carrier-protein] reductase) by forming adduct with NAD(H) needed for mycolic acid biosynthesis via affecting fatty acid synthesis II (FAS-II) pathway [4]. Similarly there are investigations reporting that INH forms complex with *β*-ketoacyl-ACP synthase (KasA) enzyme. To better understand the efficacy of INH, already several novel strategies have been adopted. For instance, genetics and biochemical approach have led to the discovery of two different target enzymes for INH within the unique type II FAS system involved in mycolic acid synthesis [5]. Similarly, quantitative proteomics reveals novel insights into INH susceptibility in mycobacteria, which is mediated by a universal stress protein. Increase in KatG levels induced by increased BCG-2013 levels underlies the phenotypic susceptibility of mycobacteria to INH [6]. In fact, the single-drug/single-target paradigm of most antibiotics may not apply to INH, as discovered by proteomic profiling, where it is believed that there are twelve INH-pyridine nucleotide metabolites, each one potentially targeting a different enzyme [7]. Even the in silico approach has shed novel insights into the mechanism of action of INH resistance by KatG gene mutations [3].

In this context, it would be of immense interest and value to comprehend the basis of resistance exhibited by MTB towards INH. Considering the fact that primary target of INH involves inhibition of mycolic acids synthesis [5, 8–11], understanding other classes of lipid compositional changes in MTB upon INH treatment may further help in identification of potential novel targets. Lipid biology has evolved to a greater extent during the last decade, primarily attributed to advancements in the sophistication of mass spectrometry (MS) instrumentation [12, 13]. Many studies have noted altered lipid metabolism associated with diseases including TB [14–21]. Therefore, study of lipid profiles by high-throughput lipidomics approach involving MS has gained significant importance in recent times. Most importantly, a comprehensive study reporting a database of MTB lipids, “Mtb LipidDB” [18], attracted our attention, which provided the impetus for us to investigate the MTB lipidome, for which we chose to apply (reverse phase) liquid chromatography coupled to electrospray ionization MS, namely, LC-ESI-MS. Consequently, the aim of this study is to compare the lipid profile of MTB in response to INH, wherein the lipid profiling is interpreted from LC-ESI-MS data. Our LC-ESI-MS data indicate that INH treatment not only causes alterations to the biosynthesis of mycolic acids that is already known, but also significantly influences the production of other lipid categories such as glycerolipids (GLs) and glycerophospholipids (GPLs), which have not been reported yet and therefore, this could be the first such report. Furthermore, we interpreted our LC-ESI-MS data using another database, MycoMass [22], whose output was compared with the output yielded from Mtb LipidDB [18] and found that the results from both the databases were similar, though a major difference was that the output from MycoMass database showed significant alterations to polyketides (PKs) category due to INH, which was not very conspicuous in the output from Mtb LipidDB. Thus, our analysis hereby provides an affirmative hint, prompting us to propose a hypothesis that INH could significantly alter the synthesis of GLs, GPLs, and PKs of MTB, in addition to that of mycolic acids. The results of this study could thus be helpful to find novel drug targets towards the treatment of MTB.

## 2. Materials and Methods 

### 2.1. Materials

INH (Sigma Aldrich), Middlebrook broth 7H9, Middlebrook agar, OADC and ADC (BD Difco), potassium chloride (KCl), dimethyl sulfoxide (DMSO) and chloroform (Fisher Scientific), methanol (Merck), Teflon capping glass vials (Borosil), Pasteur pipette, and Whatman No. 1 filter paper were the materials used.

### 2.2. Culture Conditions

MTB (clinical strain) was used for the study. Culture was maintained on Lowenstein-Jenson (LJ) slant before experiment strain was freshly streaked on agar plate after 21 days of full growth. It was again inoculated in Middlebrook 7H9 broth for 14 days as a primary (1^o^) culture. Starter culture H_37_Rv untreated or treated with INH drug at subinhibitory conc. of 0.5 *μ*g/ml was inoculated with primary (1^o^) culture of 0.1 OD_600_ in a screw cap flask containing 10 mL of Middlebrook 7H9 broth supplemented with 10% albumin/dextrose/catalase (BD Difco), 0.2% glycerol (Fischer Scientific), and 0.05% Tween-80 (Himedia) in 100 mL flasks (Schott Duran). The flasks were placed at 37°C for 14 days or till its exponential phase with agitation.

### 2.3. Extraction of Total Lipids

Cells of MTB untreated (control) and INH treated at exponential phase were used for whole cell lipid extraction by modified Folch method [21, 23]. Briefly, the MTB treated and untreated cells were harvested at 10,000 rpm for 10 minutes. Cell was homogenized in aqueous solution for 3 minutes and suspended in CHCl_3_ and CH_3_OH in ratio, 1:2. Cells were shaken well and centrifuged at 2000 rpm at 4°C for 10-15 minutes. Supernatant was transferred to another glass vial and then remaining CHCl_3_ was added and filtered through Whatman No. 1 filter paper. The extract was then washed with 0.88 % KCl to remove the nonlipid contamination, which resulted in the formation of two phases. From the two phases, the lower dense layer of chloroform containing lipid was taken by glass Pasteur pipette in 5 mL glass vial having Teflon capping. The vials were stored at -20°C until further analysis. The concentration of the extracted lipids was estimated by using a method described elsewhere [24] and 5 *μ*g/*μ*l was used for further analysis.

### 2.4. Ultraperformance Liquid Chromatography-Electrospray Ionization-Mass Spectrometry (UPLC-ESI-MS)

The samples were analyzed by LC-MS (Waters, ACQ-TQD#QBP 1152), a triple quadrupole tandem mass spectrometer in polarity switching mode. LC was done on a C18 column (100 mm x 3 mm, 2.6 *μ*m 100Å pore size) through isocratic mode of elution for 30 minutes, using 5% isopropanol, 90% methanol, and 5% ammonium acetate (5 mM, pH 6.5), at a flow rate, 0.1 ml/min. The source temperature was 120°C, the desolvation temperature was 350°C, and the cone voltage was set at 40 volts (V). The sample was introduced using an autosampler with 1 *μ*l of sample injection volume containing 5 *μ*g of lipids. Capillary voltage (i.e., spray voltage) was set to 3.50 kilo volts (kV). The data were recorded in the mass range,* m/z* 200-2000, and the data were processed within the MassLynx software, where each chromatogram was smoothened and the background was subtracted. Duplicate or triplicate LC-ESI-MS data sets were acquired and the extent of reproducibility of the data was verified. Further analysis was carried out with the reproducible LC-ESI-MS data. Analysis of the mass spectral data was performed by using a standalone software MS-LAMP, in which “Mtb LipidDB” (http://www.mrl.colostate.edu/; Sartain* et al*., 2011) [18] and the database of Lipid Metabolites and Pathways Strategy Consortium (LIPID MAPS; http://www.lipidmaps.org) are integrated [25]. Data acquired in positive ESI mode only were considered for interpretation by “Mtb LipidDB” of MS-LAMP, whereby the* m/z* values of the peaks in the mass spectra were assigned to singly protonated ion, i.e., [M+H]^+^ only, and by setting the mass window range for every search to 0.5. The same set of observed* m/z* values (that were interpreted by MS-LAMP) were analyzed using MycoMass database [22] as well, following the same search criteria; namely, only [M+H]^+^ ions were searched with mass window range set to 0.5.

### 2.5. Lipase Assay

MTB cells were grown in Middlebrook 7H9 broth in the absence (control) and presence of INH. Whole cell protein was extracted and protein concentration was determined by Lowry method as previously described [26]. Lipase activity was performed by measuring the amount of* p*-nitrophenol (*p*-NP) released from* p*-NP ester substrate with varying lengths of fatty acids. The total lipase activity was estimated using protein extract of MTB. The standard lipase activity assays were performed in 100 *μ*l reaction system consisting of a final concentration of 0.5 mM* p*-NP esters substrate and the buffer (pH 8.0) of 80 mM H_3_BO_3_, 80 mM H_3_PO_4_, 300 mM NaCl, 0.3% Triton X-100, and 20% glycerol. The reaction mixture of purified protein was incubated at 37°C for 40 minutes and the release of* p*-nitrophenol was determined spectrophotometrically at 405 nm [27, 28].

### 2.6. Criteria for Interpretation and Analysis of UPLC-ESI-MS Data

(i) Criterion based on peak intensity values: in this study, only those peak* m/z* values in the mass spectra have been considered for interpretation, whose intensities are higher than 5000 (namely, threshold value to ignore the peaks with poor signal-to-noise ratios). The peak* m/z* values selected in this manner were searched in the two databases: Mtb LipidDB and MycoMass, so as to identify the prospective lipids.

(ii) Criterion based on values of mass window range used for database search: during the course of analysis using MS-LAMP (in which Mtb LipidDB is integrated), when the mass window range for searching was set to any value less than 0.5, e.g., 0.25, very few lipids were obtained in the output from “Mtb LipidDB” (see Supplementary Material). However, while searching by using values of the mass window range > 0.5 in MS-LAMP, the mass errors (ΔM: difference between the observed or queried* m/z* value and the* m/z* value available in the database for the respective lipid) estimated for the lipids obtained in the output were found to be quite high and consequently such outputs were not considered for further interpretation. Moreover, it must be noted that the data herein have been recorded in a mass spectrometer (triple quadrupole; see Materials and Methods, Section 2.4) that is capable of offering unit resolution only, for which the mass window range = 0.5 is an optimum or suitable value to carry out searches using MS-LAMP. Likewise, for the searches performed using MycoMass also, the outputs obtained by setting the mass window range = 0.5 only were considered for further analysis. Thus, the mass errors (ΔM) of all the lipids obtained in the output herein are ≤ 0.5 and only those lipids have been considered for further interpretation. Furthermore, as mentioned in Section 2.4, all the* m/z* values have been ascribed to [M+H]^+^ only.

## 3. Results 

### 3.1. Analysis of LC-ESI-MS Data Using Mtb LipidDB Through MS-LAMP

**Figure 1:**
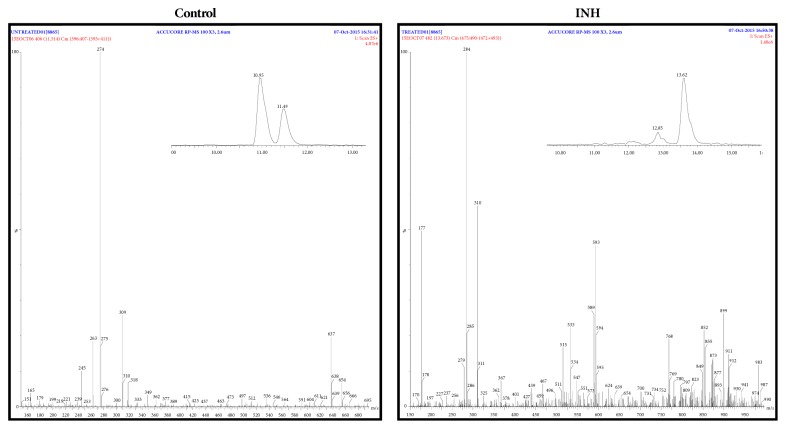
Representative mass spectra from control and INH-treated samples (see Supplementary Figures S1–S11). Inset depicts zoomed-in portion of the chromatogram showing peak at a particular retention time, corresponding to the respective mass spectrum shown here.

**Figure 2:**
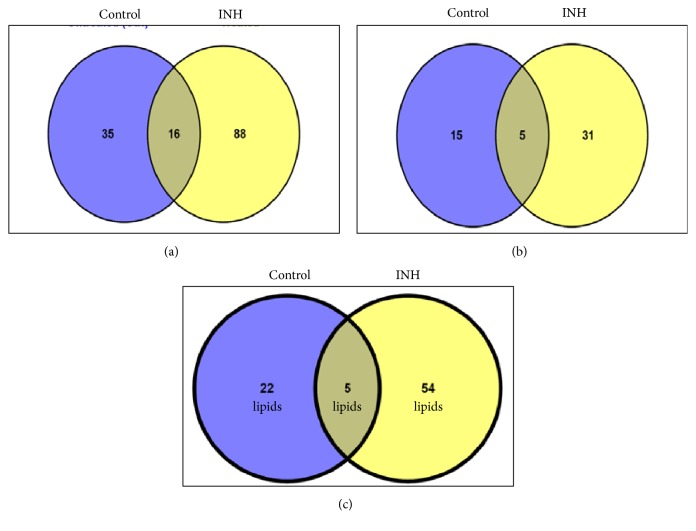
**Venn diagrammatic representation of** (a) number of* m/z* values observed in “Control” and “INH-treated” MTB samples; (b) number of* m/z* values for which lipids were identifiable in Mtb LipidDB (MS-LAMP); (c) “number of lipids” interpreted from Mtb LipidDB in “Control” and “INH-treated” MTB samples, by querying the observed* m/z* values (see Figure 2(b)) in MS-LAMP.

Figure 1 depicts representative chromatogram (inset) and mass spectra of untreated (control) and INH-treated samples. Mass spectra corresponding to different retention times in the chromatograms acquired from “control” and INH-treated samples are shown in Supplementary Material (Figures S1–S11). As already described (see Materials and Methods), only data acquired in positive ESI mode were analyzed. 51 and 104 observed* m/z* values were chosen from untreated (control) and INH-treated sample, respectively, for further interpretation, among which 16* m/z* values were observed in both the samples, which is depicted in the Venn diagram (Figure 2(a)). Strikingly, only 15 out of 35 and 31 out of 88 observed* m/z* values were interpretable by “Mtb LipidDB” (that is integrated in MS-LAMP) (Figure 2(b)). These 15 and 31* m/z* values corresponded to 22 and 54 lipids, respectively (Figure 2(c)).  Table 1 enlists the molecular formula of the potential lipids identified from “Mtb LipidDB”, corresponding to every 15 (control) and 31 (INH-treated) observed* m/z* values, which were queried in MS-LAMP, and Table 2 shows the names of the respective lipids. With regard to the 16 observed* m/z* values that are commonly detected in both “control” and “INH-treated” samples, only 5* m/z* values were interpretable by MS-LAMP, which corresponded to 5 lipids (Table 3).

### 3.2. Analysis of LC-ESI-MS Data Using MycoMass Database

We also analyzed the LC-ESI-MS data with another database, MycoMass, reported by Layre et al. 2011 [22], which contains 5399 molecules, namely, nearly twice the number of lipids present in Mtb LipidDB (2518 lipids) reported by Sartain et al. 2011 [18].  Table 4 contains the list of observed* m/z* values that were not interpretable by Mtb LipidDB, but several of those were interpretable using MycoMass database. As expected, the number of prospective lipids identified from MycoMass is greater than those resulted from Mtb LipidDB, simply because MycoMass contains nearly twofold more lipids than Mtb LipidDB.

## 4. Discussion

Lipid homeostasis holds a consequential role in MTB pathogenicity and nearly 60% of its cellular dry weight is due to lipids [18]. The lipids' diverse structural architecture and lipophobicity form an obstruction for host antibodies, sterilizers, and antimycobacterial drugs [29] and thus are beneficial for growth and survival of MTB. INH, a known anti-TB drug, targets several genes, namely,* KATG*,* NDH*,* MSH*, and* NAT*, but the primary target with dominant phenotype remains* INHA*, which encodes enoyl-Acyl-Carrier-Protein (ACP) termed as inhA reductase. The consensus on INH target demonstrates the inhibition of C26 FA elongation by FA synthase II complex, which restricts the formation of mycolic acids [9]. To obtain insights and to have a better understanding into mechanism of action of INH, we therefore decided to compare the lipidome profile of INH exposed MTB cells with the unexposed MTB cells.

Among eight lipid categories defined by LIPID MAPS (http://www.lipidmaps.org), the lipids identified thus far in MTB encompass only six categories: fatty acyls (FAs), glycerolipids (GLs), glycerophospholipids (GPLs), prenol lipids (PLs), polyketides (PKs), and saccharolipids (SLs) [18, 22]. Therefore, the results obtained from MS-LAMP covered these six categories of lipids only. As already described (*cf.* vide supra, Section 2.6), the mass window range = 0.5 was found to be an optimum value to carry out searches in MS-LAMP. Further, the data herein have been analyzed qualitatively only.

**Figure 3:**
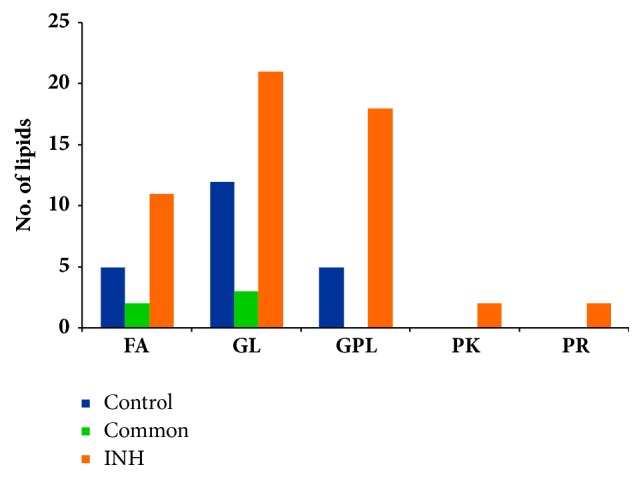
Comparison of variations in every lipid category between “Control” and “INH-treated” MTB cells: number of lipids in each category is interpreted from Mtb LipidDB [18], by querying the* m/z* values observed from the respective samples, in MS-LAMP (see Section 2.6).

Out of 22 potential lipids interpreted from the “control” sample, 5 belonged to FAs category, 12 are GLs, and 5 are GPLs (Tables 1 and 2). On the other hand, the 54 prospective lipids found from the INH-treated sample comprise 11 FAs, 21 GLs, and 18 GPLs (Figure 3 and Tables 1 and 2). Thus, the output from MS-LAMP obtained upon querying LC-ESI-MS data acquired from “control” and “INH-treated” MTB samples suggests that INH treatment triggers synthesis of not only more lipids of every lipid category (Figures 2 and 3), but also entirely different set of lipids in each lipid category that were not found in the untreated/control sample (Tables 1 and 2). Significant alterations to the lipids belonging to GL and GPL category in this investigation are notable, since studies thus far have only reported about changes happening to mycolic acids (FA category) due to INH treatment [5, 9, 10]. These different sets of lipids could be involved in the mechanism of drug resistance, which indeed opens up new vistas for more investigations.

Mycolic acid (MA) and phthiocerol dimycocerosates (DIM) belong to pathogenic FA class and it was observed that both were not detected in the presence of INH (Tables 1 and 2). DIM is one of the factors responsible for causing virulence with unresolved mechanisms; however some data showed that DIM shields MTB from oxidative stress, e.g., due to NO (nitric oxide), and modulates the production of key inflammatory cytokines such as TNF-*α*, which regulate the immunogenic responses. INH inhibits this class and makes the bacilli incapable to grow in oxidative stress [30]. It is also documented that mycolates play a significant role in cell wall permeability [31]. Therefore, INH affects the DIM level and thus makes the MTB cell more permeable. Further, it is interesting that the level of mycocerosic acid in “INH-treated” sample seems to be relatively higher as compared to the “control” sample (Tables 1 and 2). Mycocerosic acid is esterified with long-chain diol, phthiocerol, to generate phthiocerol dimycocerosate (DIM). Thus, it may be speculated that INH inhibits DIM synthesis at post-mycocerosic acid step, namely, inhibiting the esterification reaction between phthiocerol and mycocerosic acids in the biosynthetic pathway [32]. Similarly, we could detect alterations in fatty acyl chain lengths of hydroxyphthioceranic acid and phthioceranic acid, in response to INH, and found that in both classes of molecules the chain length was reduced (Tables 1 and 2). This observation prompts us to hypothesize that synthesis of sulfolipid, wherein hydroxyphthioceranic acid and phthioceranic acid are its major constituents, is affected, which are uniquely present in MTB and play critical roles in the host-pathogen interaction [32].

**Figure 4:**
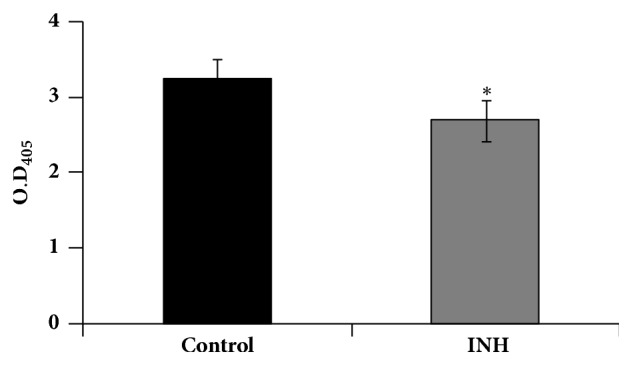
**Lipase activity in response to INH**. Mean lipase activity estimated as described in methods of three independent experiments ± SD of control and INH-treated cells are depicted as bar graph, where* y*-axis shows O.D. at 405 nm. *∗* depicts *P* value < 0.5.

The GLs category contains three different subclasses depending on the number of fatty acids attached to glycerol backbone, namely, monoacylglycerol (MG), diacylglycerol (DG), and triacylglycerol (TG). GLs can be associated with either MA [33] or non-MA too [34]. When MTB bacilli are in dormant stage or stress condition they accumulate TGs. When MTB requires energy, the TGs are catabolized by lipase enzymes that are present on the outer wall to generate FAs that could be used for energy production [21, 27]. Herein, we found that the lipase activity was significantly decreased in the presence of INH (Figure 4), which means low burning and less utilization of accumulated TGs that are required by the MTB under stress conditions, such as presence of INH in this case [35]. Towards this, the output from MS-LAMP (Tables 1 and 2; Figure 3) provides supportive evidence in that relatively higher levels of DG and TG are observed in INH-treated sample, when compared to those levels in “control” sample, wherein relatively more MG are observed (Tables 1 and 2).

GPLs form another important category of lipids, which appeared to be more responsive in presence of INH. Moreover, it is already known that virulent strains of MTB possess higher contents of GPLs [36]. Increased levels in major GPLs were detected in response to INH, which include phosphatidyl ethanolamine (PE), phosphatidylinositol (PI), phosphatidyl glycerol (PG), and phosphatidylinositol mannoside (PIMs) (Tables 1 and 2). MTB changes their lipid composition in response to various stress conditions in order to adapt to the changed environment. There are reports showing that, under INH stress, genes associated with lipid biosynthesis including GPL undergo mutation resulting in alteration of lipid composition, which further leads to the development of resistance to INH [37–39]. PIMs constitute a subclass of GPLs and are important components of the MTB cell envelope. Polar PIM species can also serve as membrane anchors for lipomannan (LM) and lipoarabinomannan (LAM). Synthesis of PIMs occurs sequentially in MTB where PIM1 helps in synthesis of PIM2 and likewise till PIM6, as revealed by extensive genetic and biochemical studies [40]. PIMs are substrates for heavy mannosylation to form LMs and additional arabinosylation to produce LAMs, which have roles in pathogenicity [21, 40]. It was found that acylated phosphatidylinositol mannosides, Ac1PIM1, were either drastically decreased or absent in presence of INH; hence we could hypothesize that acylation of PIMs is perhaps inhibited by INH and the role of acylation of PIMs towards drug resistance requires further probing.

Further, we performed comparative analysis of lipid categories identified from MycoMass database with MS-LAMP's output. The search in MycoMass database was also done in the same manner as the search was done with Mtb LipidDB using MS-LAMP, whereby the window range was set to 0.5 (see Section 2.6). As already described (*cf.* vide supra, Section 3.2), the number of lipids obtained by searching MycoMass database is greater than that yielded from Mtb LipidDB (MS-LAMP), since the number of lipids in MycoMass database is more than that of Mtb LipidDB. MycoMass database consists of maximum number of “SLs” and interestingly, Mtb LipidDB has more FAs than MycoMass (Table 5). The number of GLs in MycoMass is about thrice the number of GLs in Mtb LipidDB; however, the number of GPLs in both the databases is somewhat comparable (Table 5). Therefore, when the outputs corresponding to every lipid category resulting from each of these two databases were compared, some interesting inferences emerged.

**Figure 5:**
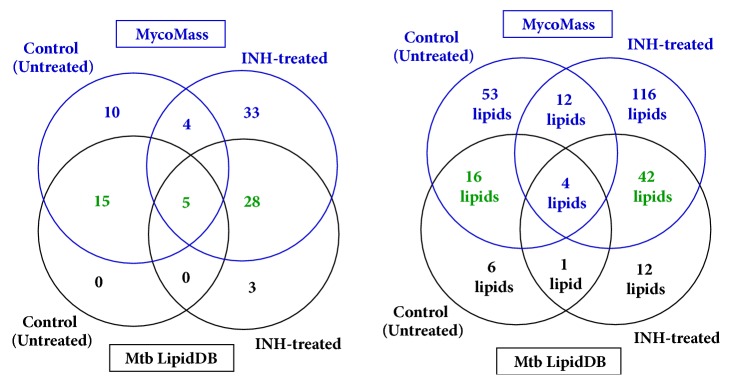
**Venn diagrammatic comparison of outputs from Mtb LipidDB (MS-LAMP) and MycoMass**. (a) No. of observed* m/z* values interpreted from or found in the respective databases. (b) No. of lipids interpreted from the observed* m/z* values, using Mtb LipidDB (MS-LAMP) and MycoMass (compare this with Figure 2); blue color depicts the output from MycoMass database, and black color is for the results obtained from Mtb LipidDB (MS-LAMP), whereas green color depicts the output obtained from both Mtb LipidDB and MycoMass databases.

Table 6 depicts the comparison of the outputs yielded from both databases, for the observed m/z values from “control” and “INH-treated” samples. Similar to the output of Mtb LipidDB (MS-LAMP), the results from the MycoMass database also indicated variations to the category of GLs and GPLs, due to INH treatment (Table 6). However, in the case of output from MycoMass database, significant alterations to polyketides (PKs) upon INH treatment too were noticeable, which was not so conspicuous in the MS-LAMP's output. This can be comprehended from Table 5, wherein it is very clear that MycoMass database contains 893 PKs, whereas only 21 PKs are present in Mtb LipidDB. Despite the significant differences between these two databases, certain observed m/z values, 15 out of 35 (control), 5 out of 16 (control and INH-treated), and 28 among 88 (INH-treated), have been interpreted by or found in both databases (Table 6). Among the 22 lipids that were interpreted from Mtb LipidDB (MS-LAMP) using the 15* m/z* values observed in the “control” sample (see Figure 2), 16 lipids are also found in MycoMass database. Furthermore, 61 out of 88* m/z* values observed in the data of “INH-treated” sample have been interpreted by MycoMass database, which correspond to a total of 158 lipids. Among these 158 lipids, 42 lipid molecules are interpretable by both databases. This suggests that the 116 lipid molecules identified from the data of “INH-treated” sample are unique to the MycoMass database. All these are pictorially illustrated in the form of Venn diagram shown in Figure 5 (also see Supplementary Tables S1–S3).

**Figure 6:**
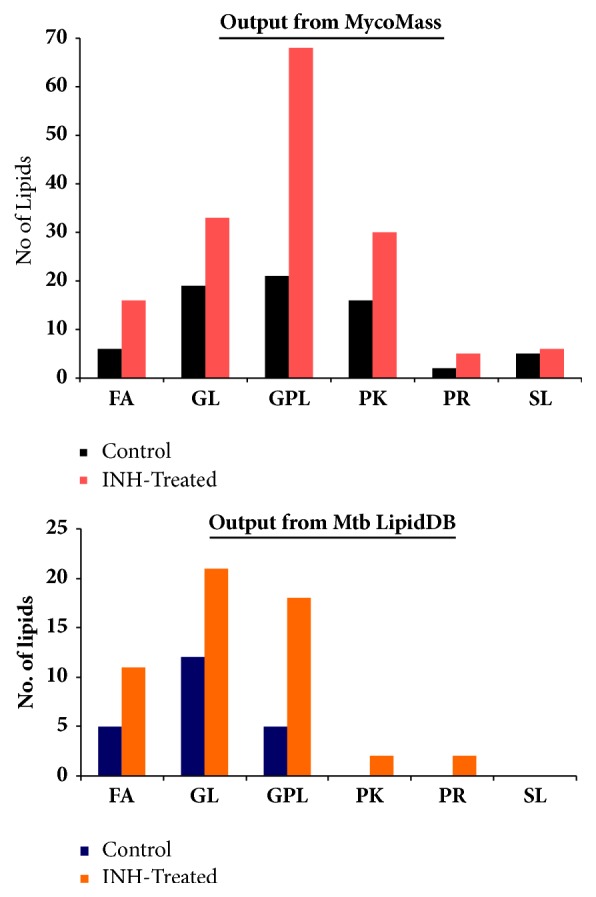
Comparison of the output obtained from MycoMass with that of Mtb LipidDB (MS-LAMP) for the “Control” and “INH-treated” MTB samples (see Section 2.6, Figure 3, and Table 6).

A closer evaluation and comparative analysis of outputs from both databases showed that 10 and 24* m/z* values observed in “control” and in “INH-treated” samples, respectively, were not found in both databases (Table 6). Likewise, it was not possible to ascribe any lipid for the 7 observed* m/z* values that were commonly found in the data from both “control” and “INH-treated” samples (Table 6). This suggests that these 41* m/z* values (Supplementary Table S4) in the mass spectral data might be due to new lipid molecules, which may have to be discovered in MTB, hinting the need to update these two databases. Alternatively, the strains of MTB utilized to create these two databases are perhaps different from the “clinical strain” that has been used in this investigation, which can be the reason for the absence of these* m/z* values in these two databases.  Figure 6 depicts bar graph representation of the data shown in Table 6, whereby the outputs from Mtb LipidDB (MS-LAMP) and MycoMass are compared.

**Figure 7:**
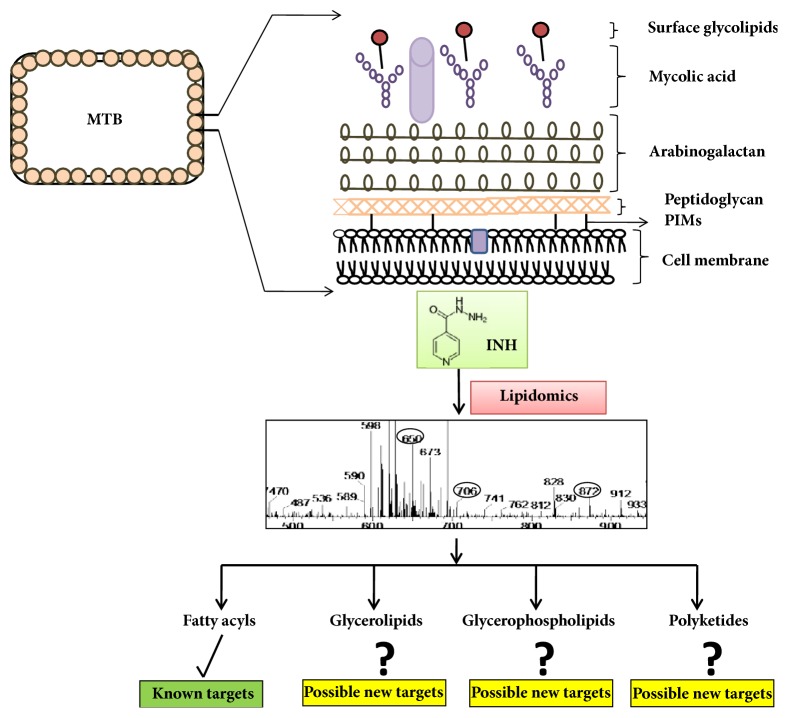
Model depicting that INH treatment alters the lipid makeup of MTB which is not limited to FAs. Variations noted with GLs, GPLs, and PKs of MTB upon INH treatment could open up new perspectives to explore novel drug targets (✓ denotes known lipid class (FA) while ? denotes possible additional lipid classes (GLs, GPLs, and PKs) affected by INH as proposed from this study).

Altogether, the observations made in this study reinforce our hypothesis that INH treatment alters the lipid makeup of MTB and this change is not limited to FAs alone. Variations noted with GLs, GPLs, and PKs of MTB upon INH treatment could indeed open up new perspectives for further investigations, thereby helping in dissecting the evolution of drug resistance mechanisms and in exploring novel drug targets (Figure 7). Though it is not clear, as to how and in what order these changes are interconnected, the data generated from this study seemingly suggest that membrane remodeling is essential for the survival of MTB under stress. The specific links between INH resistance and named lipid moieties in this study may provide diagnostic and therapeutic targets that may be instrumental to combat MDR-TB. It needs to be noted that the inferences arrived in this investigation are actually based on lipid mass fingerprinting only, which was accomplished by conventional ESI MS and database searches involving Mtb LipidDB and MycoMass. In order to validate these inferences for the sake of proving the hypothesis proposed herein, thin layer chromatography (TLC) and tandem mass spectrometry (MS/MS) of some standard compounds need to be investigated (that may be pursued in future) and the results of this study could be the basis for choosing the standard compounds. Additionally, an aspect emerging from this study, which may be useful in future, is that many of the observed* m/z* values could not be ascribed to any lipid in the currently available versions of both the databases, namely, Mtb LipidDB (MS-LAMP) as well as MycoMass, indicating that such observed* m/z* values could be due to new lipid molecules that are yet to be discovered in MTB. This means that the versions of both the databases that we have used in this study may not be adequate and perhaps require to be updated. Furthermore, it deserves special mention that several genetic and host factors do interplay and hence must be considered while designing strategies aimed to search therapeutic targets. Extensive lipidomic analysis needs to be performed, in order to pinpoint the commonalities among various MTB strains, in terms of lipidome variation. Nevertheless, this study could be a starting resource point to link the changes due to INH resistance.

## Figures and Tables

**(a):** 

**Control (Untreated)**				
**S. No.**	**Observed *m/z* [M+H]** ^**+**^	**Fatty Acyls (FAs)**	**Glycerolipids (GLs)**	**Glycerophospholipids (GPLs)**
**1**	317	-	C18 H36 O4	-

**2**	331	-	C19 H38 O4	-

**3**	409	C27 H52 O2	-	-

**4**	485	-	C30 H60 O4	C22 H45 O9 P1

**5**	497	-	-	C23 H45 O9 P1

**6**	513	-	C31 H60 O5	C24 H49 O9 P1

**7**	539	-	C33 H62 O5	-

**8**	611	-	-	C28 H51 O12 P1

**9**	626	-	C39 H76 O5	-

**10**	650	-	C41 H76 O5	-

**11**	694	C46 H92 O3	C44 H84 O5	-

**12**	706	C48 H96 O2	C45 H84 O5	-

**13**	872	-	C56 H102 O6	-

**14**	1054	-	C69 H128 O6	-

**15**	1240	C83 H162 O5, C84 H166 O4	C82 H158 O6	C65 H123 O19 P1

**(b):** 

**INH (Treated)**				
**S. No.**	**Observed *m/z* [M+H]** ^**+**^	**Fatty Acyls (FAs)**	**Glycerolipids (GLs)**	**Glycerophospholipids (GPLs)**
**1**	343	-	C20 H38 O4	-

**2**	369	C24 H48 O2 (2 nos.)	-	-

**3**	411	C27 H54 O2	-	-

**4**	413	C26 H52 O3	C25 H48 O4	-

**5**	425	C28 H56 O2	-	-

**6**	467	C31 H62 O2 (2 nos.)	-	-

**7**	483	C31 H62 O3	-	C22 H43 O9 P1

**8**	496	C33 H66 O2 (2 nos.)	-	C24 H50 N1 O7 P1

**9**	511	-	-	C24 H47 O9 P1

**10**	512	C33 H66 O3	-	-

**11**	551	-	C34 H62 O5	-

**12**	624	-	C39 H74 O5	-

**13**	700#	-	-	-

**14**	734	-	C47 H88 O5	C39 H73 O10 P1

**15**	738§	-	C46 H88 O6, C47 H92 O5	C39 H77 O10 P1

**16**	752	-	C47 H90 O6, C48 H94 O5	C40 H79 O10 P1

**17**	763	-	-	C42 H84 N1 O8 P1, C33 H63 O17 P1

**18**	780∧	-	C49 H94 O6, C50 H98 O5	C42 H83 O10 P1

**19**	794	-	C50 H96 O6, C51 H100 O5	C43 H85 O10 P1

**20**	805	-	-	C45 H90 N1 O8 P1

**21**	806	-* *-	C51 H96 O6, C52 H100 O5	C44 H85 O10 P1

**22**	850	-	C54 H104 O6, C55 H108 O5	C44 H81 O13 P1

**23**	852	-	-	C44 H83 O13 P1

**24**	854	-	-	C44 H85 O13 P1

**25**	873*∗*	-	-	-

**26**	911	-	-	C38 H71 O22 P1

**27**	930	-	C60 H112 O6	-

**28**	934	-	C60 H116 O6	-

**29**	1008	-	-	C50 H87 O18 P1

**30**	1044	-	C68 H130 O6	C52 H99 O18 P1

**31**	1058	-	C69 H132 O6	-

# C50 H82 O1: 1 prenol lipid; § C40 H81 O9 P1: 1 polyketide; ∧ C50 H83 O4 P1: 1 prenol lipid; *∗* C47 H77 N5 O10: 1 polyketide.

**(a):** 

	**Control (Untreated)**			
**S. No.**	**Observed *m/z* [M+H]** ^**+**^	**Fatty Acyls (FAs)**	**Glycerolipids (GLs)**	**Glycerophospholipids (GPLs)**
**1**	317	-	MG	-

**2**	331	-	MG	-

**3**	409	Mycolipenic acid (C27)	-	-

**4**	485	-	MG	Lyso-GP

**5**	497	-	-	Lyso-GP

**6**	513	-	DG	Lyso-GP

**7**	539	-	DG	-

**8**	611	-	-	Lyso-PI

**9**	626	-	DG	-

**10**	650	-	DG	-

**11**	694	Hydroxyphthioceranic acid (C46)	DG	-

**12**	706	Phthioceranic acid (C48)	DG	-

**13**	872	-	TG	-

**14**	1054	-	TG	-

**15**	1240	DIM-B, Methoxy-MA	TG	Ac1PIM1

MG: monoacylglycerols; DG: diacylglycerols; TG: triacylglycerols; PG: diacylglycerophosphoglycerols; PI: diacylglycerophosphoinositols; PE: diacylglycerolphosphoethanolamines; PIM: phosphatidylinositol mannosides; PIM1: phosphatidylinositol monomannosides; PIM2: phosphatidylinositol dimannoside; DIM-B: phthiodiolone dimycocerosates; MA: mycolic acids; Lyso-GP: monoacylglycerophosphoglycerols; Lyso-PI: monoacylglycerophosphoinositols; Ac1PIM1: monoacylated diacylglycerophosphoinositol monomannosides.

**(b):** 

	**INH – Treated**			
**S.No.**	**Observed *m/z* [M+H]** ^**+**^	**Fatty Acyls (FAs)**	**Glycerolipids (GLs)**	**Glycerophospholipids (GPLs)**
**1**	343	-	MG	-

**2**	369	Mycosanoic acid (C24), Mycocerosic acid (C24)	-	-

**3**	411	Mycocerosic acid (C27)	-	-

**4**	413	Mycolipanolic acid (C26)	MG	-

**5**	425	Mycocerosic acid (C28)	-	-

**6**	467	Mycocerosic acid (C31), Phthioceranic acid (C31)	-	-

**7**	483	Hydroxy phthioceranic acid (C31)	-	Lyso-GP

**8**	496	Phthioceranic acid (C33), Mycocerosic acid (C33)	-	Lyso-PE

**9**	511	-	-	Lyso-GP

**10**	512	Hydroxy phthioceranic acid (C33)	-	-

**11**	551	-	DG	-

**12**	624	-	DG	-

**13**	700 #	-	-	-

**14**	734	-	DG	PG

**15**	738 §	-	DG, TG	PG

**16**	752	-	DG, TG	PG

**17**	763	-	-	PE, Lyso-PIM1

**18**	780 ∧	-	DG, TG	PG

**19**	794	-	DG, TG	PG

**20**	805	-	-	PE

**21**	806	-	DG, TG	PG

**22**	850	-	DG, TG	PI

**23**	852	-	-	PI

**24**	854	-	-	PI

**25**	873 *∗*	-	-	-

**26**	911	-	-	Lyso-PIM2

**27**	930	-	TG	-

**28**	934	-	TG	-

**29**	1008	-	-	PIM1

**30**	1044	-	TG	PIM1

**31**	1058	-	TG	-

# decaprenol (1 prenol lipid); § phosphomycoketide (polyketide); ∧ decaprenyl phosphate (1 prenol lipid); *∗* mycobactin w/o Fe (1 polyketide).

MG: monoacylglycerols; DG: diacylglycerols; TG: triacylglycerols; PG: diacylglycerophosphoglycerols; PI: diacylglycerophosphoinositols; PE: diacylglycerolphosphoethanolamines; PIM: phosphatidylinositol mannosides; PIM1: phosphatidylinositol monomannosides; PIM2: phosphatidylinositol dimannoside; DIM-B: phthiodiolone dimycocerosates; MA: mycolic acids; Lyso-GP: monoacylglycerophosphoglycerols; Lyso-PI: monoacylglycerophosphoinositols; Lyso-PIM1: monoacylglycerophosphoinositol monomannosides; Lyso-PIM2: monoacylglycerophosphoinositol dimannosides.

**Table 3:** Molecular formula and its respective name of the lipids corresponding to the 5 observed *m/z* values out of 16, which were noted in both “control” and “INH-treated” samples, obtained from Mtb LipidDB (MS-LAMP).

**S. No.**	**Observed *m/z* [M+H]+**	**Molecular formula**	**Scientific name**	**Category**
**1**	415	C25 H50 O4	MG (RCO2H=22:0)	Glycerolipids (GL)

**2**	439	C29 H58 O2	Mycocerosic acid (C29)	Fatty Acyls (FA)

**3**	441	C28 H56 O3	Mycolipanolic acid (C28)	Fatty Acyls (FA)

**4**	598	C37 H72 O5	DG (R1CO2H+R2CO2H=34:0)	Glycerolipids (GL)

**5**	654	C41 H80 O5	DG (R1CO2H+R2CO2H=38:0)	Glycerolipids (GL)

**Table 4:** Number of lipids in each category in Mtb LipidDB and MycoMass databases.

Lipid Category	**Mtb LipidDB** *∗*	**MycoMass**∧
**FA**	**751**	**393**

**GL**	**218**	**670**

**GPL**	**1295**	**1206**

**PK**	**21**	**893**

**PR**	**7**	**38**

**SL**	**226**	**2192**

Others	0*∗*	7∧

Total	**2518**	**5399**

*∗* Others in MTB LipidDB: sterol lipids (ST) and sphingolipids (SP) (Sartain et al., 2011) [18].

∧ Others in MycoMass: 6 lipopentapeptides and 1 mycothiol (Layre et al., 2011) [22].

**Table 5:** Table showing list of observed *m/z* values, for which lipids were not identifiable in Mtb LipidDB (MS-LAMP).

**Control**	**Common**	**INH**
321	309	300

333	318	310

335	362	325^#^

338	431	339^#^

349	459^#^	342

364	520	351

432	536^#^	355^#^

449	547^#^	367^#^

457^#^	628	371^#^

470	637^#^	386

529^#^	1022	391

531^#^		393

590^#^		398^#^

620^#^		435^#^

673^#^		445^#^

828^#^		503

912^#^		515^#^

957^#^		519^#^

1001^#^		522^#^

1077		523 ^#^

		528

		533 ^#^

		537^#^

		540

		549^#^

		564^#^

		569^#^

		575^#^

		577^#^

		589^#^

		591^#^

		593^#^

		617^#^

		621^#^

		634^#^

		639^#^

		644

		686

		701^#^

		712

		726

		768

		769^#^

		770

		782

		795

		797

		803^#^

		823

		842

		849

		855

		887^#^

		893

		899^#^

		901^#^

		1036^#^

# For these observed *m/z* values, lipids were identifiable from MycoMass database; see Supplementary Tables (S1–S3).

**Table 6:** ∧Comparison of number of lipids in each category identified from Mtb LipidDB and MycoMass databases in “control” and “INH-treated” samples. Number of observed *m/z* values interpreted and not interpreted by each of these two databases in “control” and “INH-treated” samples is also compared.

Sample: *M. tb*	**Control (Untreated)**		**Common: Control**		**INH-Treated**	
			**& INH-Treated**			
No. of observed *m/z* values	**35**		**16**		**88**	

Databases	**Mtb LipidDB∧**	**MycoMass**	**Mtb LipidDB∧**	**MycoMass**	**Mtb LipidDB∧**	**MycoMass**

Lipid Categories						

**FA**	5	6	2	3	11	16

**GL**	12	19	3	5	21	33

**GPL**	5	21	0	7	18	68

**PK**	0	16	0	1	2	30

**PR**	0	2	0	0	2	5

**SL**	0	5	0	0	0	6

**Total No. of Lipids**	**22**	**69**	**5**	**16**	**54**	**158**

No. of *m/z* values found in BOTH the databases	**15**		**5**		**28**	

No. of *m/z* values found in Mtb LipidDB ONLY	**0**		**0**		**3**	

No. of *m/z* values found in MycoMass ONLY		**10**		**4**		**33**

No. of *m/z* values NOT found in both the databases	**10**		**7**		**24**	

∧See Figure 6, wherein the data shown in this table have been depicted in the form of bar graph.

## Data Availability

The data used to support the findings of this study are available from the corresponding author upon request.

## Supplementary Materials

Supplementary Materials Figures  S1–S6: mass spectra acquired through UPLC-MS (see Experimental/Materials and Methods) of extract obtained from “untreated MTB cells”. Those* m/z* values that are interpretable using Mtb LipidDB database have been encircled (see Tables 1 and 2). Figures  S6–S11: mass spectra acquired by UPLC-MS of extract obtained from “treated MTB cells”. Those* m/z* values that are interpretable using Mtb LipidDB database have been encircled (see Tables 1 and 2). Table  S1: lipids, interpreted from/found in Mtb LipidDB (MS-LAMP) and MycoMass databases, for the observed* m/z* values in the “control (untreated)” MTB sample. Table  S2: lipids interpreted from/found in Mtb LipidDB (MS-LAMP) and MycoMass databases, for the observed* m/z* values in the “INH-treated” sample. Table  S3: lipids, identified from/found in Mtb LipidDB (MS-LAMP) and MycoMass databases, for the observed* m/z* values in both Control and INH-treated MTB samples. Table  S4: observed* m/z *values not found in (not interpretable by) both Mtb LipidDB∧ and MycoMass databases*∗*.
