# Supplementary material for: Investigations into Isoniazid Treated Mycobacterium tuberculosis by Electrospray Mass Spectrometry Reveals New Insights into Its Lipid Composition
Source: J Pathog. 2018 Jun 19;2018:1454316. doi: 10.1155/2018/1454316 (PMC6029481; doi:10.1155/2018/1454316)
Supplement: Supplementary Materials — Figures S1–S6: mass spectra acquired through UPLC-MS (see Experimental/Materials and Methods) of extract obtained from “untreated MTB cells”. Those m/z values that are interpretable using Mtb LipidDB database have been encircled (see Tables 1 and 2). Figures S6–S11: mass spectra acquired by UPLC-MS of extract obtained from “treated MTB cells”. Those m/z values that are interpretable using Mtb LipidDB database have been encircled (see Tables 1 and 2). Table S1: lipids, interpreted from/found in Mtb LipidDB (MS-LAMP) and MycoMass databases, for the observed m/z values in the “control (untreated)” MTB sample. Table S2: lipids interpreted from/found in Mtb LipidDB (MS-LAMP) and MycoMass databases, for the observed m/z values in the “INH-treated” sample. Table S3: lipids, identified from/found in Mtb LipidDB (MS-LAMP) and MycoMass databases, for the observed m/z values in both Control and INH-treated MTB samples. Table S4: observed m/z values not found in (not interpretable by) both Mtb LipidDB∧ and MycoMass databases∗. [file 1454316.f1.doc]

**Supplementary Material**

**Figures S1-S6**: **Mass spectra acquired through UPLC-MS (see Experimental/Materials and Methods) of extract obtained from ‘Untreated MTB cells’. Those *m/z* values that are interpretable using M.tb LipidDB database have been encircled (see Tables 1 and 2).**

**Figure S1.**

**Figure S2.**

**Figure S3.**

**Figure S4.**

**Figure S5.**

**Figures S6-S11: Mass spectra acquired by UPLC-MS of extract obtained from ‘Treated MTB cells’ Those *m/z* values that are interpretable using Mtb LipidDB database have been encircled (see Tables 1 and 2).**

**Figure S6.**

**Figure S7.**

**Figure S8.**

**Figure S9.**

**Figure S10.**

**Figure S11.**

**Table S1:** Lipids, interpreted from / found in Mtb LipidDB (MS-LAMP) and Mycomass databases, for the observed *m/z* values in the "Control (Untreated)" MTB sample.

|  |  | **Mtb LipidDB^ (MS-LAMP)** | | | **MycoMass database** | | | | |
| --- | --- | --- | --- | --- | --- | --- | --- | --- | --- |
| **S. No.** | **m/z (obs.)** | **Mol. Formula** | **Lipid** | **[M+H]+** | **Mol. Formula** | | **Lipid** | | **[M+H]+** |
| **1** | **317** | **C18 H36 O4** | **GL** (MG) | **317.269185** | **C18 H36 O4** | | **GL** (MAG) | | **317.26864** |
| **2** | **331** | **C19 H38 O4** | **GL** (MG) | **331.284835** | **C19 H38 O4** | | **GL** (MAG) | | **331.28429** |
| **3** | **409** | **C27 H52 O2** | **FA** (Mycolipenic acid) | **409.404555** | **C27 H52 O2** | | **FA** (Unsaturated) | | **409.40401** |
|  |  |  |  |  | **C27 H52 O2** | | **FA** (Phthienoic acid) | | **409.40401** |
|  |  |  |  |  | C19 H37 O7 P1 | | GPL (LPA) | | 409.23497 |
| **4** | 457 | - | - |  | C20 H41 O9 P1 | | GPL (LPG) | | 457.2561 |
|  |  |  |  |  | C27 H52 O5 | | GL (DAG) | | 457.38875 |
|  |  |  |  |  | C28 H56 O4 | | GL (MAG) | | 457.42514 |
| **5** | **485** | **C22 H45 O9 P1** | **GPL** (Lyso-GP) | **485.287946** | **C22 H45 O9 P1** | | **GPL** (LPG) | | **485.2874** |
|  |  | **C30 H60 O4** | **GL** (MG) | **485.456985** | **C30 H60 O4** | | **GL** (MAG) | | **485.45644** |
|  |  |  |  |  | C29 H56 O5 | | GL (DAG) | | 485.42005 |
| **6** | **497** | **C23 H45 O9 P1** | **GPL** (Lyso-GP) | **497.287946** | **C23 H45 O9 P1** | | **GPL** (LPG) | | **497.2874** |
|  |  |  |  |  | C30 H56 O5 | | GL (DAG) | | 497.42005 |
|  |  |  |  |  | C31 H60 O4 | | GL (MAG) | | 497.45644 |
| **7** | **513** | **C24 H49 O9 P1** | **GPL** (Lyso-GP) | **513.319246** | **C24 H49 O9 P1** | | **GPL** (LPG) | | **513.3187** |
|  |  | **C31 H60 O5** | **GL** (DG) | **513.4519** | **C31 H60 O5** | | **GL** (DAG) | | **513.45135** |
|  |  |  |  |  | C32 H64 O4 | | GL (MAG) | | 513.48774 |
|  |  |  |  |  | C36 H48 O2 | | Prenol (MK-5) | | 513.37271 |
| **8** | 529 | - | - |  | C22 H41 O12 P1 | | GPL (LPI) | | 529.24084 |
| **9** | 531 | - | - |  | C22 H43 O12 P1 | | GPL (LPI) | | 531.25649 |
| **10** | **539** | **C33 H62 O5** | GL (DG) | **539.46755** | **C33 H62 O5** | | GL (DAG) | | **539.467** |
|  |  |  |  |  | C26 H51 O9 P1 | | GPL (LPG) | | 539.33435 |
| **11** | 590 | - | - |  | C30 H56 O8 N1 P1 | | GPL (PE) | | 590.38163 |
| **12** | **611** | C28 H51 O12 P1 | GPL (Lyso-PI) | 611.319641 | C30 H59 O10 P1 | | GPL (PG) | | 611.39186 |
|  |  |  |  |  | C31 H63 O9 P1 | | GPL (LPG) | | 611.42825 |
| **13** | 620 | - | - |  | C32 H62 O8 N1 P1 | | GPL (PE) | | 620.42858 |
|  |  |  |  |  | C33 H66 O7 N1 P1 | | GPL (LPE) | | 620.46497 |
| **14** | **626** | **C39 H76 O5** | **GL** (DG) | **625.5771** | **C39 H76 O5** | | GL (DAG) | | 625.57655 |
| **15** | **650** | C41 H76 O5 | GL (DG) | 649.5771 | C34 H68 O8 N1 P1 | | GPL (PE) | | 650.47553 |
| **16** | 673 | - | - |  | C33 H46 O10 N5 | | 3 PKs (DDCM) | | 673.33175 |
|  |  |  |  |  | C37 H69 O8 P1 | | GPL (PA) | | 673.48028 |
| **17** | **694** | **C46 H92 O3** | **FA** (Branched FA) | **693.71247** | **C46 H92 O3** | | **FA** (Hydroxy Phthioceranic acid) | | **693.71192** |
|  |  | **C44 H84 O5** | **GL** (DG) | **693.6397** | **C44 H84 O5** | | **GL** (DAG) | | **693.63915** |
|  |  |  |  |  | C37 H73 O9 P1 | | GPL (LPG) | | 693.5065 |
| **18** | **706** | **C45 H84 O5** | **GL** (DG) | **705.6397** | **C45 H84 O5** | | **GL** (DAG) | | **705.63915** |
|  |  | C48 H96 O2 | FA (Branched FA) | 705.748855 | C44 H80 O6 | | GL (TAG) | | 705.60277 |
|  |  | **Mtb LipidDB^ (MS-LAMP)** | | | **MycoMass database** | | | | |
| **S. No.** | **m/z (obs.)** | **Mol. Formula** | **Lipid** | **[M+H]+** | **Mol. Formula** | **Lipid** | | **[M+H]+** | |
| **19** | 828 | - | - |  | C45 H72 O9 N5 | 3 PK s (MDMB) | | 827.54028 | |
|  |  | - | - |  | C46 H76 O8 N5 | 3 PK s (DDMB) | | 827.57667 | |
|  |  | - | - |  | C48 H91 O8 P1 | GPL (PA) | | 827.65243 | |
| **20** | **872** | **C56 H102 O6** | **GL (TG)** | **871.775465** | **C56 H102 O6** | **GL** (TAG) | | **871.77492** | |
|  |  |  |  |  | C51 H99 O8 P1 | GPL (PA) | | 871.71503 | |
|  |  |  |  |  | C48 H86 O13 | SL (DAT) | | 871.61412 | |
|  |  |  |  |  | C48 H80 O9 N5 | 3 PKs (MDMB) | | 871.60288 | |
|  |  |  |  |  | C47 H76 O10 N5 | 3 PKs (MB) | | 871.5665 | |
| **21** | 912 | - | - |  | C54 H103 O8 P1 | GPL (PA) | | 911.74633 | |
|  |  |  |  |  | C48 H94 O15 | GL (Dglc DAG) | | 911.66655 | |
|  |  |  |  |  | C55 H91 O8 P1 | PR (DPPR) | | 911.65243 | |
|  |  |  |  |  | C51 H84 O9 N5 | PK (MDMB) | | 911.63418 | |
|  |  |  |  |  | C50 H86 O15 | SL (TAT) | | 911.60904 | |
| **22** | 957 | - | - |  | C56 H110 O8 N1 P1 | GPL (PE) | | 956.80418 | |
| **23** | 1001 | - | - |  | C59 H118 O8 N1 P1 | GPL (PE) | | 1000.86678 | |
| **24** | **1054** | C69 H128 O6 | GL (TG) | 1053.978915 | C72 H140 O3 | FA (alpha-MA) | | 1054.08752 | |
| **25** | **1240** | **C82 H158 O6** | GL (TG) | **1240.213665** | **C82 H158 O6** | GL (TAG) | | **1240.21312** | |
|  |  | **C83 H162 O5 #** | **FA (DIM-B)** | 1240.25005 | **C83 H162 O5 #** | **GL (GroMM)** | | 1240.2495 | |
|  |  | **C84 H166 O4** | **FA** (Methoxy-MA) | **1240.286435** | **C84 H166 O4** | **FA** (MA) | | **1240.28589** | |
|  |  | C65 H123 O19 P1 | GPL (Ac1PIM1) | 1239.847446 | C78 H158 O9 | FA (GMM) | | 1240.19786 | |
|  |  |  |  |  | C74 H142 O13 | SL (DAT) | | 1240.05232 | |
|  |  |  |  |  | C73 H138 O14 | SL (TAT) | | 1240.01594 | |
|  |  |  |  |  | C74 H142 O13 | SL (TMM) | | 1240.05232 | |
| **Total No. of Lipids** | |  | **22** |  |  | **69** | |  | |

^ Sartain et al [18]

Note: ⮚ Those observed m/z values, for which same lipid molecular formula are obtained from both the databases, are shown in red colored bold faced font. Those same molecular formula found in both the databases are bold faced. The m/z values that are bold faced in black color are found in both the databases, but correspond to different molecular formula.

⮚ The molecular formula and lipids that are highlighted in yellow color are found only in Mtb LipidDB (MS-LAMP).

# Interestingly, the molecular formula, C83 H162 O5 corresponded to Fatty Acyl in Mtb Lipid DB (MS-LAMP), whereas the same molecular formula was found to be a Glycerolipid (GroMM) in MycoMass database.

(**Table S2**: Lipids interpreted from/found in Mtb LipidDB (MS-LAMP) and Mycomass databases, for the observed *m/z* values in the "INH-Treated" sample.

|  |  | **Mtb LipidDB^ (MS-LAMP)** | | | | **MycoMass database** | | | |
| --- | --- | --- | --- | --- | --- | --- | --- | --- | --- |
| **S.No.** | **m/z (obs.)** | **Mol. Formula** | **Lipid** | | **[M+H]+** | **Mol. Formula** | **Lipid** | | **[M+H]+** |
| 1 | 325 | - | - | |  | C21 H40 O2 | FA (Unsaturated) | | 325.31011 |
| 2 | 339 | - | - | |  | C22 H42 O2 | FA (Unsaturated) | | 339.32576 |
| 3 | **343** | **C20 H38 O4** | GL (MG) | | **343.284835** | **C20 H38 O4** | GL (MAG) | | **343.28429** |
| 4 | 355 | - | - | |  | C23 H46 O2 | FA (Mycosanoic acid) | | 355.35706 |
|  |  |  |  | |  | C15 H31 O7 P1 | GPL (LPA) | | 355.18802 |
| 5 | 367 | - | - | |  | C24 H46 O2 | FA (Unsaturated) | | 367.35706 |
|  |  |  |  | |  | C16 H31 O7 P1 | GPL (LPA) | | **367.18802** |
| 6 | **369** | **C24 H48 O2** | FA (Mycosanoic acid) | | **369.373255** | **C24 H48 O2** | FA (Mycosanoic acid) | | **369.37271** |
|  |  | **C24 H48 O2** | FA (Mycocerosic acid) | | **369.373255** | **C24 H48 O2** ^ | FA (Straight chain) ^ | | **369.37271** |
|  |  |  |  | |  | C16 H33 O7 P1 | GPL (LPA) | | 369.20367 |
| 7 | 371 | - | - | |  | C22 H42 O4 | GL (MAG) | | 371.31559 |
| 8 | 398 | - | - | |  | C17 H36 O7 N1 P1 | GPL (LPE) | | 398.23022 |
| 9 | **411** | **C27 H54 O2** | FA (Mycocerosic acid) | | **411.420205** | **C27 H54 O2** | FA (Mycocerosic acid) | | **411.41966** |
|  |  |  |  | |  | **C27 H54 O2** * | FA (Branched) * | | **411.41966** |
|  |  |  |  | |  | C19 H39 O7 P1 | GPL (LPA) | | 411.25062 |
| 10 | **413** | **C25 H48 O4** | GL (MG) | | **413.363085** | **C25 H48 O4** | GL (MAG) | | **413.36254** |
|  |  | C26 H52 O3 | FA (Mycolipanolic acid) | | 413.39947 |  |  | |  |
| 11 | **425** | **C28 H56 O2** | FA (Mycocerosic acid) | | **425.435855** | **C28 H56 O2** | FA (Mycocerosic acid) | | **425.43531** |
|  |  |  |  | |  | **C28 H56 O2** # | FA (Phthioceranic acid) # | | **425.43531** |
|  |  |  |  | |  | **C28 H56 O2** # | FA (Straight chain) # | | **425.43531** |
|  |  |  |  | |  | C20 H41 O7 P1 | GPL (LPA) | | 425.26627 |
| 12 | 435 | - | - | |  | C21 H39 O7 P1 | GPL (LPA) | | 435.25062 |
| 13 | 445 | - | - | |  | C31 H40 O2 | PR (Menaquinone,  MK-4) | | 445.31011 |
| 14 | **467** | **C31 H62 O2** | FA (Mycocerosic acid) | | **467.482805** | **C31 H62 O2** | FA (Mycocerosic acid) | | **467.48226** |
|  |  | **C31 H62 O2** | FA (Phthioceranic acid) | | **467.482805** | **C31 H62 O2** | FA (Phthioceranic acid) | | **467.48226** |
|  |  |  |  | |  | **C31 H62 O2** § | FA (Branched) § | | **467.48226** |
|  |  |  |  | |  | C28 H50 O5 | GL (DAG) | | 467.3731 |
|  |  |  |  | |  | C23 H47 O7 P1 | GPL (LPA) | | 467.31322 |
| 15 | **483** | **C31 H62 O3** | FA (Hyd. Phthioceranic acid) | | **483.47772** | **C31 H62 O3** | FA (Hyd. Phthioceranic acid) | | **483.47717** |
|  |  |  |  | |  | C29 H54 O5 | GL (DAG) | | 483.4044 |
|  |  |  |  | |  | C30 H58 O4 | GL (MAG) | | 483.44079 |
|  |  |  |  | |  | C25 H39 O7 P1 | GPL (LPA) | | 483.25062 |
|  |  | **C22 H43 O9 P1** | GPL (Lyso-GP) | | **483.272296** | **C22 H43 O9 P1** | GPL (LPG) | | **483.27175** |
| 16 | **496** | **C24 H50 N1 O7 P1** | GPL (Lyso-GP) | | **496.340315** | **C24 H50 O7 N1 P1** | GPL (LPE) | | **496.33977** |
|  |  | C33 H66 O2 | FA (Phthioceranic acid) | | 496.514105 |  |  | |  |
|  |  | C33 H66 O2 | FA (Mycocerosic acid) | | 496.514105 |  |  | |  |
|  |  | **Mtb LipidDB^ (MS-LAMP)** | | | | **MycoMass database** | | | |
| **S. No.** | **m/z (obs.)** | **Mol. Formula** | **Lipid** | | **[M+H]+** | **Mol. Formula** | **Lipid** | | **[M+H]+** |
| 17 | **511** | **C24 H47 O9 P1** | GPL (Lyso-GP) | | **511.303596** | **C24 H47 O9 P1** | GPL (LPG) | | **511.30305** |
|  |  |  |  | |  | C32 H62 O4 | GL (MAG) | | 511.47209 |
|  |  |  |  | |  | C31 H58 O5 | GL (DAG) | | 511.4357 |
| 18 | 512 | C33 H66 O3 | FA (Hyd. Phthioceranic acid) | | 511.50902 | - | - | |  |
| 19 | 515 | - | - | |  | C21 H39 O12 P1 | GPL (LPI) | | 515.22519 |
|  |  |  |  | |  | C36 H50 O2 | PR (Menaquinone,  MK-5) | | 515.38836 |
| 20 | 519 | - | - | |  | C30 H63 O4 P1 | PK (PM: Phosphomycoketide) | | 519.45367 |
| 21 | 522 | - | - | |  | C26 H52 O7 N1 P1 | GPL (LPE) | | 522.35542 |
| 22 | 523 | - | - | |  | C32 H58 O5 | GL (DAG) | | 523.4357 |
|  |  |  |  | |  | C27 H55 O7 P1 | GPL (LPA) | | 523.37582 |
| 23 | 533 | - | - | |  | C27 H49 O8 P1 | GPL (PA) | | 533.32378 |
|  |  |  |  | |  | C31 H65 O4 P1 | PK (PM: Phosphomycoketide) | | 533.46932 |
| 24 | 537 | - | - | |  | C33 H60 O5 | GL (DAG) | | 537.45135 |
|  |  |  |  | |  | C40 H56 | PR (b-carotene) | | 537.44548 |
|  |  |  |  | |  | C27 H53 O8 P1 | GPL (PA) | | 537.35508 |
|  |  |  |  | |  | C28 H57 O7 P1 | GPL (LPA) | | 537.39147 |
| 25 | 549 | - | - | |  | C28 H53 O8 P1 | GPL (PA) | | 549.35508 |
|  |  |  |  | |  | C29 H57 O7 P1 | GPL (LPA) | | 549.39147 |
| 26 | **551** | **C34 H62 O5** | GL (DG) | | **551.46755** | **C34 H62 O5** | GL (DAG) | | **551.467** |
|  |  |  |  | |  | C28 H55 O8 P1 | GPL (PA) | | 551.37073 |
|  |  |  |  | |  | C29 H59 O7 P1 | GPL (LPA) | | 551.40712 |
| 27 | 564 | - | - | |  | C29 H58 O7 N1 P1 | GPL (LPE) | | 564.40237 |
| 28 | 569 | - | - | |  | C28 H57 O9 P1 | GPL (LPG) | | 569.3813 |
| 29 | 575 | - | - | |  | C30 H55 O8 P1 | GPL (PA) | | 575.37073 |
| 30 | 577 | - | - | |  | C30 H57 O8 P1 | GPL (PA) | | 577.38638 |
|  |  |  |  | |  | C31 H61 O7 P1 | GPL (LPA) | | 577.42277 |
| 31 | 589 | - | - | |  | C31 H57 O8 P1 | GPL (PA) | | 589.38638 |
| 32 | 591 | - | - | |  | C31 H59 O8 P1 | GPL (PA) | | 591.40203 |
|  |  |  |  | |  | C32 H63 O7 P1 | GPL (LPA) | | 591.43842 |
| 33 | 593 | - | - | |  | C31 H61 O8 P1 | GPL (PA) | | 593.41768 |
|  |  |  |  | |  | C32 H65 O7 P1 | GPL (LPA) | | 593.45407 |
| 34 | 617 | - | - | |  | C33 H61 O8 P1 | GPL (PA) | | 617.41768 |
| 35 | 621 | - | - | |  | C31 H57 O10 P1 | GPL (PG) | | 621.37621 |
|  |  |  |  | |  | C34 H69 O7 P1 | GPL (LPA) | | 621.48537 |
|  |  |  |  | |  | C33 H65 O8 P1 | GPL (PA) | | 621.44898 |
| 36 | **624** | **C39 H74 O5** | GL (DG) | | **623.56145** | **C39 H74 O5** | GL (DAG) | | **623.5609** |
| 37 | 634 | - | - | |  | C33 H64 O8 N1P1 | GPL (LPE) | | 634.44423 |
|  |  |  |  | |  | C34 H68 O7 N1 P1 | GPL (LPE) | | 634.48062 |
|  |  | **Mtb LipidDB^ (MS-LAMP)** | | | | **MycoMass database** | | | |
| **S. No.** | **m/z (obs.)** | **Mol. Formula** | | **Lipid** | **[M+H]+** | **Mol. Formula** | | **Lipid** | **[M+H]+** |
| **38** | 639 | - | | - |  | C32 H63 O10 P1 | | GPL (PG) | 639.42316 |
|  |  |  | |  |  | C33 H67 O9 P1 | | GPL (LPG) | 639.45955 |
| **39** | 700 | C50 H82 O1 | | Prenol Lipid | 699.64439 | - | | - |  |
| **40** | 701 | - | | - |  | C35 H50 O10 N5 | | **5** PKs (DDCM) | 701.36305 |
| **41** | **734** | **C47 H88 O5** | | GL (DG) | **733.671** | **C47 H88 O5** | | GL (DAG) | **733.67045** |
|  |  | **C39 H73 O10 P1** | | GPL (PG) | **733.501961** | **C39 H73 O10 P1** | | GPL (PG) | **733.50141** |
|  |  |  | |  |  | C41 H81 O8 P1 | | GPL (PA) | 733.57418 |
| **42** | **738** | **C46 H88 O6** | | GL (DG) | **737.7023** | **C46 H88 O6** | | GL (TAG) | **737.66537** |
|  |  | **C47 H92 O5** | | GL (TG) | **737.665915** | **C47 H92 O5** | | GL (DAG) | **737.70175** |
|  |  | **C39 H77 O10 P1** | | GPL (PG) | **737.533261** | **C39 H77 O10 P1** | | GPL (PG) | **737.53271** |
|  |  | **C40 H81 O9 P1** | | Polyketide ü | **737.569646** | **C40 H81 O9 P1** | | PK (MPM) | **737.5691** |
| **43** | **752** | **C47 H90 O6** | | GL (DG) | **751.71795** | **C47 H90 O6** | | GL (TAG) | **751.68102** |
|  |  | **C48 H94 O5** | | GL (TG) | **751.681566** | **C48 H94 O5** | | GL (DAG) | **751.7174** |
|  |  | **C40 H79 O10 P1** | | GPL (PG) | **751.548911** | **C40 H79 O10 P1** | | GPL (PG) | **751.54836** |
|  |  |  | |  |  | C41 H83 O9 P1 | | PK (MPM) | 751.58475 |
| **44** | **763** | **C42 H84 N1 O8 P1** | | GPL (PE) | **762.60128** | **C42 H84 N1 O8 P1** | | GPL (PE) | **762.60073** |
|  |  | C33 H63 O17 P1 | | GPL (Lyso-PIM1) | 763.388116 | C37 H56 O12 N5 | | **6** PKs (CM) | 763.39983 |
| **45** | 769 | - | | - |  | C38 H73 O13 P1 | | GPL (PI) | 769.48616 |
|  |  |  | |  |  | C45 H69 O8 P1 | | GPL (PA) | 769.48028 |
|  |  |  | |  |  | C42 H66 O8 N5 | | PK (DDMB) | 769.49842 |
| **46** | **780** | **C49 H94 O6** | | GL (DG) | **779.74925** | **C49 H94 O6** | | GL (TAG) | **779.71232** |
|  |  | **C50 H98 O5** | | GL (TG) | **779.705** | **C50 H98 O5** | | GL (DAG) | **779.7487** |
|  |  | **C42 H83 O10 P1** | | GPL (PG) | **779.610721** | **C42 H83 O10 P1** | | GPL (PG) | **779.57966** |
|  |  | C50 H83 O4 P1 | | Prenol Lipid ü | 779.610721 | C43 H87 O9 P1 | | PK (MPM) | 779.61605 |
| **47** | **794** | **C50 H96 O6** | | GL (DG) | **793.7649** | **C50 H96 O6** | | GL (TAG) | **793.72797** |
|  |  | **C51 H100 O5** | | GL (TG) | **793.728515** | **C51 H100 O5** | | GL (DAG) | **793.76435** |
|  |  | **C43 H85 O10 P1** | | GPL (PG) | **793.595861** | **C43 H85 O10 P1** | | GPL (PG) | **793.59531** |
|  |  |  | |  |  | C44 H89 O9 P1 | | PK (MPM) | 793.6317 |
| **48** | 803 | - | | - |  | C40 H60 O12 N5 | | **3** PKs (CM) | 803.43113 |
|  |  |  | |  |  | C41 H64 O11 N5 | | **2** PKs (MDCM) | 803.46751 |
|  |  |  | |  |  | C45 H88 O8 N1 P1 | | GPL (PE) | 802.63203 |
| **49** | **805** | **C45 H90 N1 O8 P1** | | GPL (PE) | **804.64823** | **C45 H90 N1 O8 P1** | | GPL (LPE) | **804.64768** |
|  |  |  | |  |  | C40 H62 O12 N5 | | **3** PKs (CM) | 805.44678 |
| **50** | **806** | **C52 H100 O5** | | GL (TG) | **805.728515** | **C52 H100 O5** | | GL (DAG) | **805.76435** |
|  |  | **C51 H96 O6** | | GL (DG) | **805.7649** | **C51 H96 O6** | | GL (TAG) | **805.72797** |
|  |  | **C44 H85 O10 P1** | | GPL (PG) | **805.595861** | **C44 H85 O10 P1** | | GPL (PG) | **805.59531** |
|  |  |  | |  |  | C43 H80 O13 | | SL (DAT) | 805.56717 |
|  |  | **Mtb LipidDB^ (MS-LAMP)** | | | | **MycoMass database** | | | |
| **S. No.** | **m/z (obs.)** | **Mol. Formula** | **Lipid** | | **[M+H]+** | **Mol. Formula** | **Lipid** | | **[M+H]+** |
| 51 | **850** | **C55 H108 O5** | GL (TG) | | **849.791115** | **C55 H108 O5** | GL (DAG) | | **849.82695** |
|  |  | **C54 H104 O6** | GL (DG) | | **849.8275** | **C54 H104 O6** | GL (TAG) | | **849.79057** |
|  |  |  |  | |  | C47 H93 O10 P1 | GPL (PG) | | 849.65791 |
|  |  | **C44 H81 O13 P1** | GPL (PI) | | **849.549306** | **C44 H81 O13 P1** | GPL (PI) | | **849.54876** |
| 52 | **852** | **C44 H83 O13 P1** | GPL (PI) | | **851.564956** | **C44 H83 O13 P1** | GPL (PI) | | **851.56441** |
| 53 | **854** | **C44 H85 O13 P1** | GPL (PI) | | **853.580606** | **C44 H85 O13 P1** | GPL (PI) | | **853.58006** |
|  |  |  |  | |  | C48 H78 O8 N5 | **3** PKs (DDMB) | | 853.59232 |
|  |  |  |  | |  | C61 H88 O2 | PR (Menaquinone,  MK-10) | | 853.68571 |
| 54 | **873** | C47 H77 N5 O10 | Polyketide | | 872.57487 | C50 H98 O8 N1 P1 | GPL (PE) | | 872.71028 |
| 55 | 887 | - | - | |  | C51 H100 O8 N1 P1 | GPL (PE) | | 886.72593 |
| 56 | 899 | - | - | |  | C52 H100 O8 N1 P1 | GPL (PE) | | 898.72593 |
| 57 | 901 | - | - | |  | C52 H102 O8 N1 P1 | GPL (PE) | | 900.74158 |
| 58 | 911 | C38 H71 O22 P1 | GPL (Lyso-PIM2) | | 911.470685 | - | - | |  |
| 59 | **930** | **C60 H112 O6** | GL (TG) | | **929.853715** | **C60 H112 O6** | GL (TAG) | | **929.85317** |
|  |  |  |  | |  | C61 H116 O5 | GL (DAG) | | 929.88955 |
|  |  |  |  | |  | C55 H109 O8 P1 | GPL (PA) | | 929.79328 |
|  |  |  |  | |  | C53 H101 O10 P1 | GPL (PG) | | 929.72051 |
|  |  |  |  | |  | C51 H86 O10 N5 | PK (MB) | | 929.64475 |
|  |  |  |  | |  | C52 H96 O13 | SL (DAT) | | 929.69237 |
| 60 | **934** | **C60 H116 O6** | GL (TG) | | **933.885015** | **C60 H116 O6** | GL (TAG) | | **933.88447** |
|  |  |  |  | |  | C61 H120 O5 | GL (GroMM) | | 933.92085 |
|  |  |  |  | |  | C61 H120 O5 | GL (DAG) | | 933.92085 |
|  |  |  |  | |  | C53 H105 O10 P1 | GPL (PG) | | 933.75181 |
|  |  |  |  | |  | C50 H93 O13 P1 | GPL (PI) | | 933.64266 |
| 61 | **1008** | C50 H87 O18 P1 | GPL (PIM1) | | 1007.57083 | C55 H107 O13 P1 | GPL (PI) | | 1007.75221 |
| 62 | 1036 | - | - | |  | C57 H111 O13 P1 | GPL (PI) | | 1035.78351 |
| 63 | **1044** | **C68 H130 O6** | GL (TG) | | **1043.99457** | **C68 H130 O6** | GL (TAG) | | **1043.99402** |
|  |  | C52 H99 O18 P1 | GPL (PIM1) | | 1043.66473 | C69 H134 O5 | FA (MA) | | 1044.0304 |
|  |  |  |  | |  | C69 H134 O5 | GL (DAG) | | 1044.0304 |
|  |  |  |  | |  | C61 H119 O10 P1 | GPL (PG) | | 1043.86136 |
|  |  |  |  | |  | C60 H114 O13 | SL (DAT) | | 1043.83322 |
|  |  |  |  | |  | C59 H110 O14 | SL (TAT) | | 1043.79684 |
| 64 | **1058** | **C69 H132 O6** | GL (TG) | | **1058.01022** | **C69 H132 O6** | GL (TAG) | | **1058.00967** |
|  |  |  |  | |  | C62 H121 O10 P1 | GPL (PG) | | 1057.87701 |
|  |  |  |  | |  | C76 H112 O2 | PR (Menaquinone,  MK-13) | | 1057.87351 |
|  |  |  |  | |  | C61 H116 O13 | SL (DAT) | | 1057.84887 |
|  |  |  |  | |  | C60 H112 O14 | SL (TAT) | | 1057.81249 |
| **Total No. of Lipids** | |  | **54 lipids** | |  |  | **158 lipids** | |  |

Note: ⮚ Those observed m/z values, for which same lipid molecular formula are obtained from both the databases, are shown in red colored bold faced font. Those same molecular formula found in both the databases are bold faced. The m/z values that are bold faced in black color are found in both the databases, but correspond to different molecular formula.

⮚ The molecular formula and lipids that are highlighted in yellow color are found only in Mtb LipidDB (MS-LAMP).

**Table S3**: Lipids, identified from / found in Mtb LipidDB (MS-LAMP) and Mycomass databases, for the observed *m/z* values in both Control & INH-Treated MTB samples.

|  | | | | | | |  | | |  | | |
| --- | --- | --- | --- | --- | --- | --- | --- | --- | --- | --- | --- | --- |
|  | | | | |  |  | |  | |  | | |
|  |  |  | **MS-LAMP^** | | | | | | **MycoMass database** | | | |
|  | **S. No.** | **m/z (obs.)** | **Mol. Formula** | **Lipid** | | | **[M+H]+** | | **Mol. Formula** | | **Lipid** | **[M+H]+** |
|  | **1** | **415** | **C25 H50 O4** | GL (MG) | | | 415.378735 | | **C25 H50 O4** | | GL (MG) | 415.37819 |
|  | **2** | **439** | **C29 H58 O2** | FA (Mycocerosic acid) | | | 439.451505 | | **C29 H58 O2** | | FA (Mycocerosic acid) | 439.45096 |
|  |  |  |  |  | | |  | | **C29 H58 O2** | | FA (Branched FA) | 439.45096 |
|  |  |  |  |  | | |  | | C21 H43 O7 P1 | | GPL (LPA) | 439.28192 |
|  | **3** | **441** | **C28 H56 O3** | FA (Mycolipanolic acid) | | | 441.43077 | | **C28 H56 O3** | | FA (Hyd. Phthioceranic acid) | 441.43022 |
|  |  |  |  |  | | |  | | C27 H52 O4 | | GL (MG) | 441.39384 |
|  |  |  |  |  | | |  | | C19 H37 O9 P1 | | GPL (LPG) | 441.2248 |
|  | **4** | 459 | - | - | | |  | | C23 H39 O7 P1 | | GPL (LPA) | 459.25062 |
|  | **5** | 536 | - | - | | |  | | C27 H54 O7 N1 P1 | | GPL (LPE) | 536.37107 |
|  | **6** | 547 | - | - | | |  | | C28 H51 O8 P1 | | GPL (PA) | 547.33943 |
|  |  |  |  |  | | |  | | C32 H67 O4 P1 | | PK (PM) | 547.48497 |
|  | **7** | **598** | **C37 H72 O5** | GL (DG) | | | **597.5458** | | **C37 H72 O5** | | GL (DAG) | **597.54525** |
|  | **8** | 637 | - | - | | |  | | C32 H61 O10 P1 | | GPL (PG) | 637.40751 |
|  |  |  |  |  | | |  | | C33 H65 O9 P1 | | GPL (PG) | 637.4439 |
|  | **9** | **654** | **C41 H80 O5** | GL (DG) | | | **653.6084** | | **C41 H80 O5** | | GL (DAG) | **653.60785** |
|  |  |  |  |  | | |  | | **C40 H76 O6** | | GL (TAG) | **653.57147** |
|  |  | **Total** |  | **5 lipids** | | |  | |  | | **16 lipids** |  |

**Table S4**: Observed *m/z* values not found in (not interpretable by) both Mtb LipidDB^ as well as MycoMass databases*.

| S. No. | **Control** | **Control & INH-Treated** | **INH-Treated** |
| --- | --- | --- | --- |
| 1 | 321 | 309 | 300 |
| 2 | 333 | 318 | 310 |
| 3 | 335 | 362 | 342 |
| 4 | 338 | 431 | 351 |
| 5 | 349 | 520 | 386 |
| 6 | 364 | 628 | 391 |
| 7 | 432 | 1022 | 393 |
| 8 | 449 |  | 503 |
| 9 | 470 |  | 528 |
| 10 |  |  | 540 |
| 11 |  |  | 644 |
| 12 |  |  | 686 |
| 13 |  |  | 712 |
| 14 | 1077 |  | 726 |
| 15 |  |  | 768 |
| 16 |  |  | 770 |
| 17 |  |  | 782 |
| 18 |  |  | 795 |
| 19 |  |  | 797 |
| 20 |  |  | 823 |
| 21 |  |  | 842 |
| 22 |  |  | 849 |
| 23 |  |  | 855 |
| 24 |  |  | 893 |

^ Sartain et al., 2011 [18]; Sabareesh & Singh., 2013 [25]

*Layre et al., 2011 [22]

|  |  |  |  |  |  |  |  |
| --- | --- | --- | --- | --- | --- | --- | --- |
